# ELL2 Influences Transcription Elongation, Splicing, Ig Secretion and Growth

**Published:** 2019-07-12

**Authors:** Anthony Ghobrial, Nathaniel Flick, Ryan Daly, Malcolm Hoffman, Christine Milcarek

**Affiliations:** Department of Immunology, School of Medicine, University of Pittsburgh, Pittsburgh, PA 15213, USA

**Keywords:** Gene expression, Homology, Tumour, Proto-oncogene, Immunology

## Abstract

ELL2 was previously discovered as a member of the Super Elongation Complex. It is involved in driving the maturation of B cells to plasma cells through shifting patterns of RNA processing, favoring generation of the secretory form of heavy chain immunoglobulin (IgH) associated with plasma cells. ELL2 influences the expression and splicing patterns of more than 4,000 genes in antibody secreting cells. The ELL2 gene has been implicated in cancers such as multiple myeloma and salivary gland carcinoma. A member of the ELL family (ELL1) was recently proven to act as an E3 ubiquitin ligase to known proto-oncogene, c-Myc, through a highly conserved cysteine residue in the C-terminal CEYLH region. Comparison of sequence homology shows this region is conserved between the three members of the ELL family, leading us to hypothesize that the other two ELLs (2 and 3) could serve the same role. In this review, we summarize what is known about ELL2 with respect to its role in driving B cell to plasma cell differentiation as well as its potential role in tumor suppression.

## Introduction

Maturation of peripheral B cells after exposure to cognate antigen or bacterial lipopolysaccharide (LPS) often drives the cells to the plasma blast stage and further to differentiation into antibody secreting cells (ASC); these cells are generally short lived. A number of genes involved in this process have been studied extensively, including blimp-1 (Prdm1), IRF4, and Xbp-1 [1].

The action of blimp-1 as either a positive or negative transcriptional regulator has been recently reviewed [2]. None of these conventional, initiation-focused, transcription factors directly influences the massive changes in RNA levels and processing that take place when Ig heavy chain mRNA levels increase as much as 50-fold and the gene is alternatively processed to produce the secreted form of the Ig. ELL2, the eleven-nineteen lysine-rich leukemia gene, which is induced in the transition to ASCs, influences the Ig heavy chain genes directly to change the pattern of RNA processing from the membrane specific form in B cells, to the secretory-specific mRNA in ASCs. ELL2 also enhances transcription through-put from the IgH gene resulting in more mRNA [3, 4].

**Figure 1:**
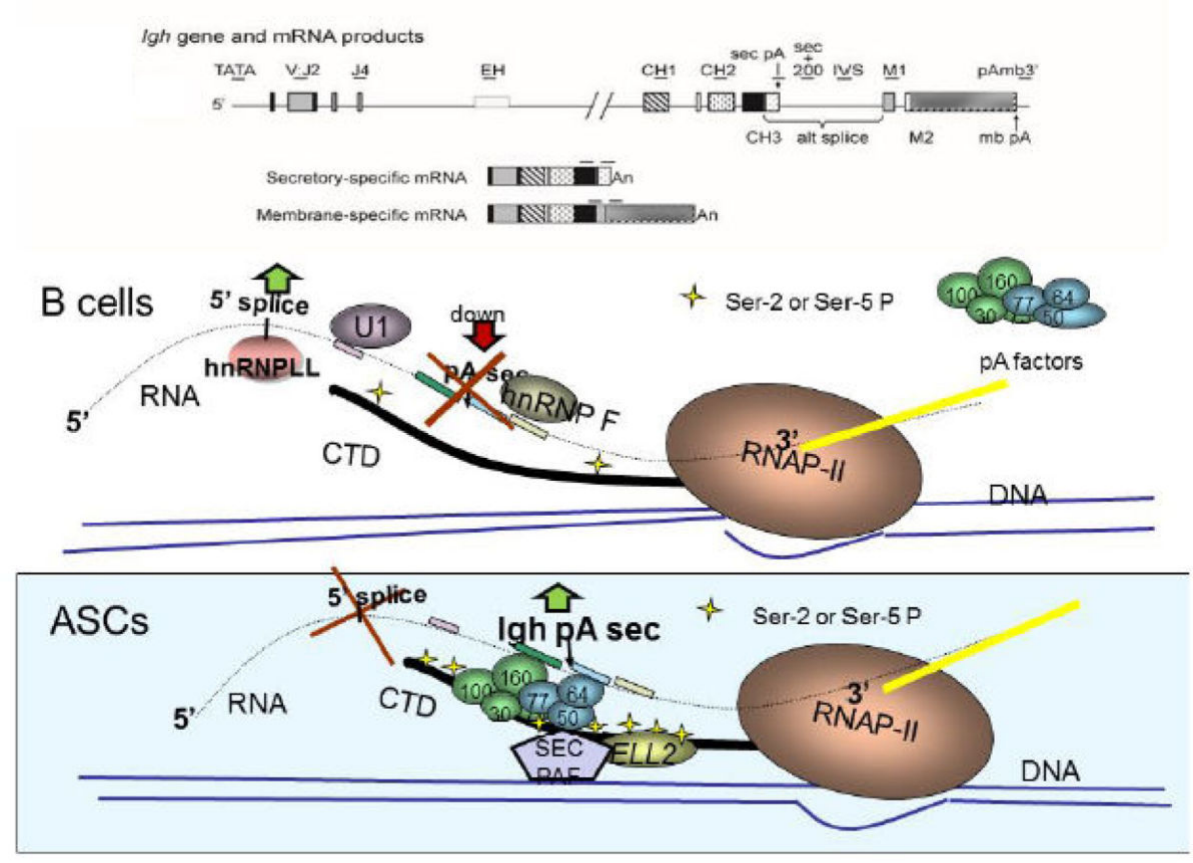
Proposed role of ELL2 in the processing of Ig mRNA. In B cells the polymerase lacks the SEC while in antibody secreting cells the polymerase is equipped with all the factors necessary to use the first aka secretion-specific poly(A) site and produce secretory-specific IgH mRNA.

Knocking out ELL2 in mouse B cells results in reduced Ig secretion and alteration in the expression of the unfolded protein response genes [4]. Figure 1 depicts an IgG gene, the RNA products made from it, and the postulated role of ELL2 in altering Ig mRNA processing. In B cells the polymerase lacks the SEC while in antibody secreting cells the polymerase is equipped with all the factors necessary to use the first aka secretion-specific poly(A) site and produce secretory-specific IgH mRNA.

## Experimental

### Role of ELL2

We have previously reviewed the expression and function of ELL2 in the transition to ASCs where it causes the polymerase “to travel to the beat of different drum” [5,6]; we briefly summarize what is known here, so that we can put recent advances in context. RNA polymerase II (RNAP II) pauses after initiation in a number of genes, including IgH. Additional factors in a so-called “super elongation complex” or SEC, are generally required to kick-start the paused polymerase. The SEC is composed of pTEFb (cdk9 and cyclin T), Aff4, ENL, and one of the ELLs. The ELL 1, 2, and 3 genes are very similar but are differentially expressed as we have previously described [5]. While the stoichiometry of the SEC has not been rigorous determined, it appears that the ELL molecule is present once per complex.

Therefore, large amounts of ELL2 in ASCs could drive increased SEC formation and decrease pausing. The pTEFb is responsible for the ser-5 phosphorylation of the repeated sequence at the carboxyl-terminal domain of RNAP II.

The SEC has been shown to travel with the polymerase down the gene (3’-ward) along with a polymerase-associated complex called Paf. The Paf recruits the polyadenylation factors (like CstFs, CPSFs, etc.) so that the entire RNAP II, SEC, Paf, polyadenylation-factor complex travels towards the 3’ end of IgH gene in ASCs. We have shown that there is high ser-5P on RNAP II and loading of polyadenylation factors on the 5’end of the IgH gene in ASCs [7]. The factors in the complex recognize the first/secretory-specific polyA site and utilize it, thereby precluding the use of the 5’ splice site to the exons that specify the membrane-binding region of IgH down-stream (see Figure 1 for the model).

When we recently examined which genes were most severely influenced by the loss of ELL2 in B cells in the conditional knockout mouse we found the following: the ASC signature genes; N-glycan biosynthesis and processing genes; Cell cycle genes: Golgi and ER processing genes [8,9].

We found that ER-stress responses of Ire-1 phosphorylation and the accompanying RIDD (regulated Ire1-dependent mRNA decay pathways) were induced before ELL2 up-regulation and the major wave of Ig secretion. We found that when ELL2 is maximally induced, there are large-scale changes in the splicing patterns in ASCs as compared with B cells; as many as 4,000 genes are influenced in their splicing patterns. Even genes whose mRNA abundances did not change had altered splicing patterns. Interestingly, genome wide association studies (GWAS) of altered IgG N-glycosylation phenotypes uncovered ELL2 as a significant gene for IgG glycosylation in humans [10] confirming our observations.

### Role of ELL2 in cancer

ELL1 was discovered as one of many fusion-partners in multiple lineage leukemia (MLL); many other potential MLL fusion partners are also components of the SEC. ELL2 and ELL3 were isolated independently because of their homology to ELL1. ELL1 and ELL3 proteins have been shown to associate with p53 and based on sequence identity ELL2 might be expected to also associate with p53 [11]. Salivary gland carcinomas are rare malignancies with unknown etiology.

Meta-analysis of the non-Hispanic white and Hispanic cohorts with salivary gland tumors identified a genome-wide significant single nucleotide polymorphisms in ELL2 [12]. Meanwhile there are 20 pleiotropic variant groups composed of 33 variants including novel pleiotropic variants rs3777204 and rs56219066 that are located in the ELL2 gene and associated with cancer susceptibility [13].

### Role of ELL2 in Multiple myeloma

Multiple myeloma is a disease characterized by formation of tumors in the bone marrow resulting from uncontrolled growth of a terminally differentiated plasma cell. It accounts for 1.6% of United States cancer cases [14]. Previous studies have implicated ELL2 as a susceptibility gene for multiple myeloma [15], with a specific non-coding mutation (rs3888189-C) being associated with the disruption of MAF/G/K transcription factor binding as well as the reduced expression of both ELL2 itself and ribosomal genes in ASCs. The rs3888189-C allele has also been linked to diseases of cell types resembling ASCs, such as salivary gland carcinoma [15].

The Thr298Ala missense variant of ELL2 was identified as a multiple myeloma risk; non-cancerous individuals bearing that mutation show reduced levels of IgA or IgG and an increased risk of bacterial meningitis [16]. The correlation with cancer and an ELL2 mutation may relate to p53 associations, or to a reduced ability to ubiquitinate Myc1, leading to enhanced growth. Aberrant regulation and elongation of ELL2 target cell cycle genes, as we have shown, would also be expected to perturb cell growth.

### ELL and Myc

**Figure 2:**
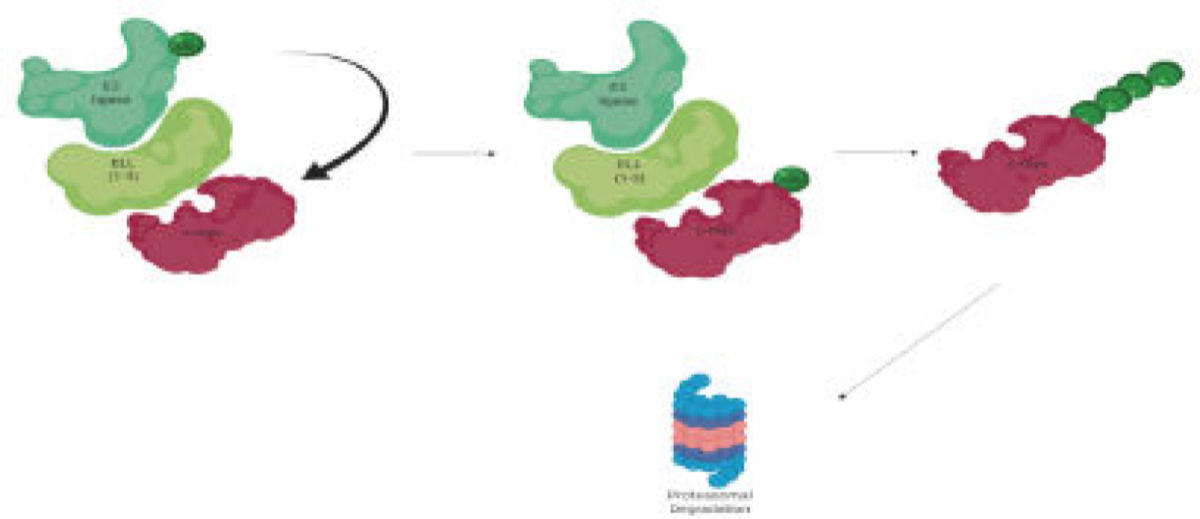
Proposed interaction between ELLs and c-Myc through E3 ubiquitination pathway. ELL1 serves as an E3 ubiquitin ligase of c-Myc, serving as a mediator between the E2 ligase and the c-Myc substrate so that ubiquitin tagging can occur, leading to proteasomal degradation of the ubiquitinated c-Myc.

ELL1 targets c-Myc for ubiquitination and proteasomal degradation; as a consequence ELL1 inhibits tumor growth [17]. Previous studies established ELL1 as an E3 ubiquitin ligase of the transcription factor and known proto-oncogene, c-Myc (Figure 2).

**Figure 3:**
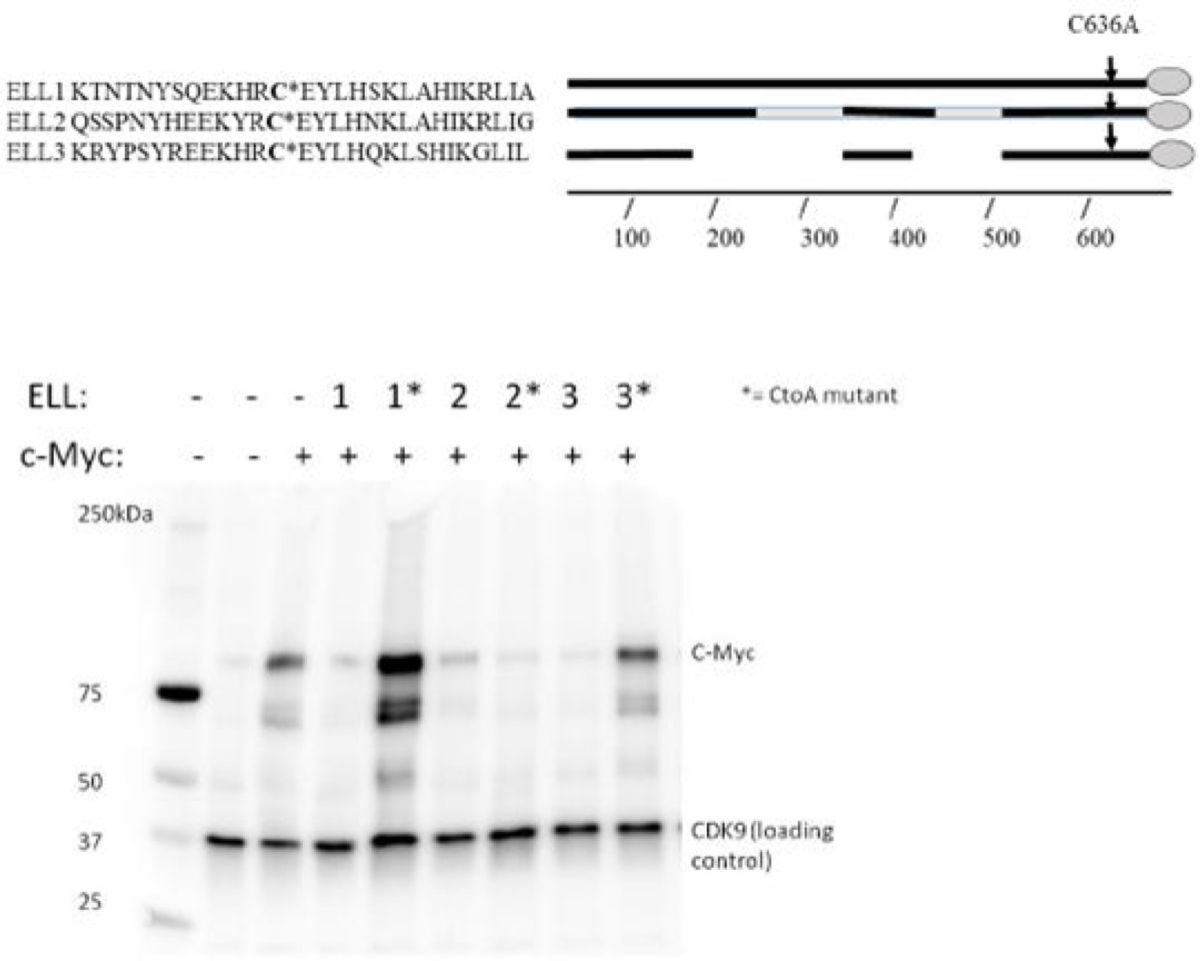
Protein sequence homology between ELLs 1, 2, and 3. The asterisk marks the Cysteine previously determined to be key in facilitating ELL1’s role as an E3 ligase. Conservation is maintained between all 3 ELLs as noted by the arrow.

Shared homology in the CEYLH region near the carboxyl-terminus (Figure 3), previously determined to be the active site of such interaction, suggest that ELL2 and 3 could share this function with ELL1 in facilitating the ubiquitination and subsequent degradation of c-Myc. To study this relationship, HEK293T cells were transfected with c-Myc, or both c-Myc and the ELLs, to observe if over-expression of the ELL proteins results in c-Myc decline/degradation. Western blots from such transfections show that addition of any of the three respective ELLs resulted in decreased C-Myc levels in comparison to the C-Myc single transfects. Mutation of the Cysteine to Alanine in the CEYLH region of ELLs 1 and 3 restore Myc levels. Interestingly, the ELL2 CtoA mutation retained the ability to degrade c-Myc.

These results suggest that ELLs 2 and 3 serve a similar role to ELL in the Ubiquitin tagging of c-Myc for proteasomal degradation. The results also suggest that ELL3, like ELL, relies on the conserved C residue in the CEYLH region for this interaction to take place while ELL2 may have another site of action, as yet undetermined.

## Results

Analysis of c-Myc levels in the presence of Wildtype and C636AMutant ELLs. For all 3 respective ELLs, dual transfections with c-Myc showed decline in c-Myc intensity relative to c-Myc single transfection alone. For ELLs 1 and 3, c-Myc levels were either all or partially restored through the C636A mutation, suggesting this site is key for E3 ligase activity. The C636A mutation to ELL2 did not restore c-Myc levels, suggesting this site may not play a role in ELL2’s proposed E3 ligase activity. (c-Myc (D84C12) Rabbit mAb (Cell signaling product #5605S) was used to detect c-Myc, while Cdk9 (D7) Mouse mAb (Santa Cruz product #sc-13130) was used to detect Cdk9).

## Discussion and Conclusion

Turning the gene for ELL2 on during the differentiation of B cells to antibody secreting cells not only changes the splicing pattern of the IgH gene, but also influences many other genes in the cell, both in the quantity but also in the quality of the mRNA. It has been estimated that genes with multiple exons can produce 4–5 different mRNA products, some encoding proteins with different NH2 or Carboxyl or internal exons; ELL2 facilitates this shift in expression through influencing splicing. When ELL2 is induced to high levels it can also accelerate the growth of the B cells to the plasmablast stage by turning on cell cycle genes. But, ELL2 expression can carry the seeds of the plasmablast’s own impermanence by destroying c-Myc and thereby halting growth. In order to proceed to the terminal stage of long-term survival for antibody secreting cells other growth factors and their receptors must be necessary. This interaction of ELL and c-Myc protein leading to its destruction may explain how tumors arise with mutated versions of ELL that cannot hold c-Myc action in check. Further work on this topic may yield new insights into the link between splicing and cancer. How ELL2 differs from ELL1 or ELL3 in its action on c-Myc may also be of interest.
